# Transfer Function Analysis of the Longitudinal Motion of the Common Carotid Artery Wall

**DOI:** 10.3389/fphys.2016.00651

**Published:** 2016-12-27

**Authors:** Heikki Yli-Ollila, Mika P. Tarvainen, Tomi P. Laitinen, Tiina M. Laitinen

**Affiliations:** ^1^Department of Clinical Physiology and Nuclear Medicine, Kuopio University Hospital (KYS)Kuopio, Finland; ^2^Department of Applied Physics, University of Eastern Finland (UEF)Kuopio, Finland; ^3^Department of Radiology, Kanta-Häme Central HospitalHämeenlinna, Finland; ^4^Department of Clinical Physiology and Nuclear Medicine, University of Eastern Finland (UEF)Kuopio, Finland

**Keywords:** adventitia, arterial stiffness, blood pressure, frequency space, intima-media, motion tracking, power spectrum, ultrasound imaging

## Abstract

The longitudinal motion of the carotid wall is a potential new measure of arterial stiffness. Despite the over decade long research on the subject, the driving force and the specific longitudinal kinetics of the carotid wall has remained unclear. In this study, a transfer function analysis with 20 healthy subjects is presented to derive how the energy from the blood pressure moves the innermost arterial wall longitudinally and how the kinetic energy is then transferred to the outermost arterial layer. The power spectrums display that the main kinetic energy of the longitudinal motion is on band 0–3 Hz with a peak on the 1.1 Hz frequency. There is a large variation among the individuals, how the energy from the blood pressure transfers into the longitudinal motion of the arterial wall since the main direction of the longitudinal motion varies individually and because early arterial stiffening potentially has an effect on the time characteristics of the energy transfer. The energy transfer from the innermost to the outermost wall layer is more straightforward: on average, a 17% of the longitudinal amplitude is lost and an 18.9 ms delay is visible on the 1.0 Hz frequency.

## Introduction

Longitudinal motion of the carotid artery wall is an interesting phenomenon with partly unknown origin. Ultrasound imaging has proven to be a good technique to measure the phenomenon (Golemati et al., 2003; Persson et al., 2003; Svedlund and Gan, 2011a; Zahnd et al., 2011b; Yli-Ollila et al., 2013; Albinsson et al., 2014). Most of the longitudinal shear is believed to be between the media and the adventitia layers of the arterial wall (Nilsson et al., 2010). Due to the low contrast of the media layer in the ultrasound images, intima and media layers are usually measured as a one complex (Cinthio et al., 2006). The amplitude of the longitudinal motion of the carotid wall has been shown to be liked to well-known stiffness indices: a direct correlation to carotid artery distensibility and an inverse correlation to pulse wave velocity has been found (Taivainen et al., 2015). In addition, the reduction of the amplitude of the longitudinal motion of the carotid intima layer has been linked to aging, hypertension, diabetes, plaque burden in the common carotid artery and the risk of myocardial ischemia and cardiovascular disease (Svedlund and Gan, 2011b; Svedlund et al., 2011; Zahnd et al., 2011a, 2012; Tat et al., 2015). In animal models, the reduction of the amplitude of the longitudinal carotid wall motion has been associated with plaque burden in brachiocephalic artery as well as with the total cholesterol level in blood (Svedlund and Gan, 2011b). However, recent results display that focusing on the amplitude of the longitudinal motion may be a restrictive approach, the fine structure of the motion has also potential to reveal the status of the vascular system (Taivainen et al., 2015; Yli-Ollila et al., 2015, 2016).

Transfer function analysis is a widely used tool in electronics to reveal linear relationship between two connected signals. In the field of cardiovascular research, the transfer function analysis has been used in studies of vascular function to model aortic pressure curve by measuring the pressure only from superficial arteries (Karamanoglu and Feneley, 1996; Segers et al., 2000). In addition, the transfer function analysis has been used to investigate the relationship between arterial blood pressure and cerebral blood flow (Kuo et al., 1998), to identify high-grade carotid stenosis with impaired cerebral autoregulation (Hu et al., 1999) and to study the effect of aging and hypertension on cerebral artery blood flow (Lipsitz et al., 2000). Nevertheless, the transfer function analysis has not previously been used to investigate the longitudinal motion of the artery wall.

The aim of this study is to use the transfer function analysis to characterize the longitudinal motion between the intima-media complex and the adventitia layer as well as blood pressure and intima-media complex in healthy subjects in detail. Using referential stiffness measurements, the spectral characteristics of the transfer function of the longitudinal motion are investigated as potential indicators of early arterial stiffening.

## Materials and methods

### Subjects and study protocol

Twenty healthy, non-smoking volunteers with no history of cardiac diseases participated in the study. Ten of the volunteers were men and 10 were women. One male subject was omitted from the analysis because during measurement a left bundle branch block was found and the subject could not be presented as a healthy volunteer. The clinical characteristics of the study population have been presented in Table 1. First, the volunteer was positioned in the supine position and ECG-electrodes were attached on the chest. Then the volunteer was allowed to rest for 10 min and the blood pressure was measured from the left upper arm using an automatic blood pressure monitor (Omron, M4-I, Matsusaka, Japan). Immediately after the blood pressure measurement, an anatomical B-mode ultrasound video was imaged from the left common carotid artery using 18 MHz linear transducer (Philips EPIQ 7, L18-5 transducer, Best, The Netherlands). The ultrasound transducer was handled by an experienced physician and positioned on the carotid artery, thus the bifurcation was merely visible on the right side of the ultrasound image. The size of the ultrasound frame was 1.25 × 1.5 cm (width × height) and the used imaging frame rate was 85 Hz. A total of 5-min acquisition was achieved by collecting a set of thirteen 10 s ultrasound videos. After the ultrasound acquisition, the blood pressure was measured again and an average of the two blood pressure measurements was calculated.

**Table 1:** **Clinical characteristics of the study population**.

	**Age (years)**	**Height (cm)**	**Weight (kg)**	**Brachial SBP/DBP (mmHg)**	**Carotid SBP/DBP (mmHg)**	**Heart rate (Hz) [bpm]**
Avg	29	174	69	115/68	121/69	1.09 [65]
*SD*	7	10	10	11/6	13/6	0.16 [10]
Range	19–49	155–189	52–85	96–135/60–84	96–141/60–84	0.75–1.36 [45–82]

*Avg, average; SD, standard deviation; SBP, systolic blood pressure; DBP, diastolic blood pressure. n = 19 (9 men, 10 women)*.

Finally an applanation tonometry measurement was performed on the subject using SphygmoCor (version 9, AtCor Medical Inc., Itasca, IL, USA). In the applanation tonometry measurement, a pen like pressure probe (SPT-301B, Millar instruments, Houston, TX, USA) was positioned on the radial artery of the subject. The SphygmoCor system measures the average pressure waveform of the radial artery and by using in-build transfer function analysis it computes the pressure waveform in the aorta and in the carotid artery. A widely used arterial stiffness measure augmentation index adjusted for the heart rate of 75 beats per minute (Aix@75) was obtained from the system and SphygmoCor system was also used to transform the systolic and diastolic upper arm pressures (averages of the two measurements) into carotid pressures.

All volunteers were prohibited from drinking coffee for 2 h before the experiment. Fully informed written consent was obtained from each participant and the Ethics Committee of the University of Eastern Finland and Kuopio University Hospital approved the used study protocol.

### Motion tracking and blood pressure curve calculation

**Figure 1:**
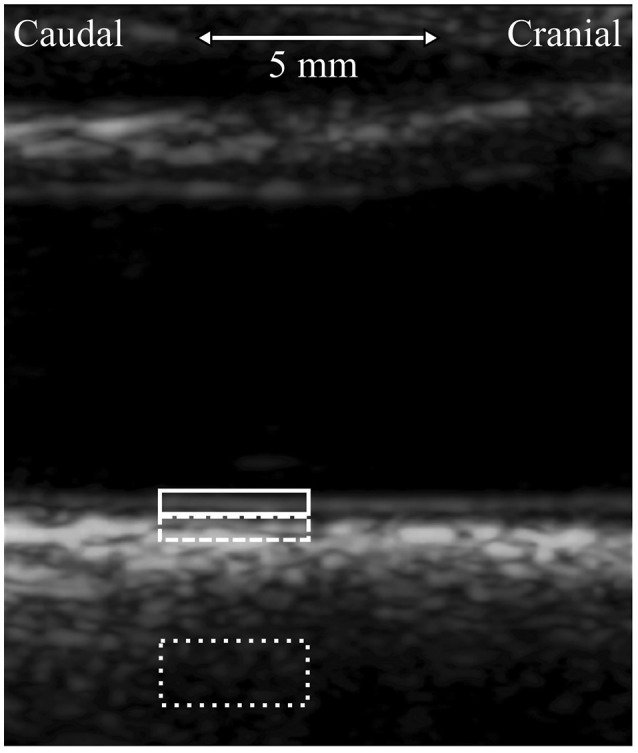
**Longitudinal view of the left common carotid artery, imaged 5 mm to caudal direction from the carotid bifurcation**. The typical locations of the regions of interest (ROI) used in the tracking of the longitudinal motion of the carotid wall are presented with white color boxes. ROI of the intima-media complex, solid line; ROI of the adventitia layer, dashed line; ROI of the surrounding tissue, dotted line.

The longitudinal motion tracking from the B-mode videos was performed in Matlab (R2013b, The MathWorks Inc., Natick, MA, USA) using our in-house-build motion tracking program, including contrast optimization (Yli-Ollila et al., 2013). One region of interest (ROI) was positioned on the intima-media complex of the far carotid wall and another ROI was positioned on the adventitia layer and third referential ROI on surrounding tissues behind the carotid artery, see Figure 1. To simultaneously track the diameter change of the artery for blood pressure curve calculations, additional ROIs were positioned on the near and far carotid wall. The sizes of the ROIs were otherwise freely selectable but the widths of the ROIs were forced to be the same. The average size of the intima-media, adventitia and surrounding tissue ROIs were (width × height) 2.58 × 0.33 mm, 2.58 × 0.30 mm, and 2.58 × 1.15 mm, respectively. The average sizes of the ROIs used for the diameter tracking were 2.58 × 0.97 mm for the near wall and 2.58 × 1.83 mm for the far wall.

To ensure accurate measurement, the motion tracking was done in heartbeat long sequences and the accuracy of the motion tracking was monitored by an experienced physicist after every cardiac cycle. If there were problems (i.e., swallowing) in the longitudinal motion traces, the observed heartbeat long signal was cut out from the transfer function analysis. The motion of the surrounding tissue was eliminated from the final longitudinal intima-media and adventitia traces, i.e., the recorded longitudinal motion of the surrounding tissue ROI was subtracted from the recorded longitudinal motion of the intima-media and adventitia ROIs.

The carotid blood pressure signal was computed from the carotid diameter change curve. The values of the diameter curve were linearly changed to be the blood pressure values subject wise by computing the average diameter curve and setting the systolic and diastolic diameter to average systolic and diastolic blood pressure, obtained from the blood pressure measurements before and after the ultrasound scanning. The linear relationship between blood pressure and artery diameter has been found previously (Barnett et al., 1961; Patel et al., 1963; Sugawara et al., 2000; Giannattasio et al., 2008).

### Longitudinal motion parameters

**Figure 2:**
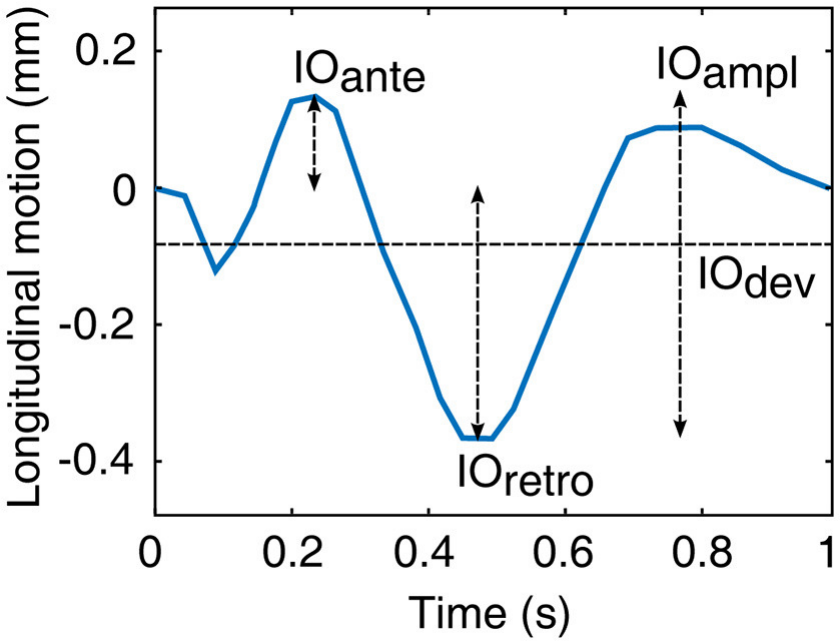
**Illustration of the motion parameters derived from the longitudinal motion curve between the intima-media complex of the common carotid artery wall and surrounding tissues (origo)**. IO_ampl_, peak-to-peak amplitude; IO_ante_, antegrade component of the peak-to-peak amplitude; IO_retro_, retrograde component of the peak-to-peak amplitude; IO_dev_, average deviation from the baseline.

From the 5-min longitudinal motion tracings, an average, heartbeat long longitudinal motion signal was computed for every subject. From this average motion curve, four longitudinal motion parameters were computed: IO_ampl_, peak-to-peak amplitude of the longitudinal motion of intima-media; IO_ante_, antegrade (in the direction of the main blood flow) component of the longitudinal amplitude; IO_retro_, retrograde (against the direction of the main blood flow) component of the longitudinal amplitude; IO_dev_, average deviation of the longitudinal trace from the baseline during a heartbeat. The baseline is the longitudinal position of the intima-media at the moment of ECG R-spike. IO_dev_ describes the main direction of the longitudinal motion: minus sign represents motion into the retrograde direction and plus sign represents motion into the antegrade direction. See Figure 2 for illustration of the longitudinal motion parameters.

### Transfer function analysis

Two different transfer functions were computed in this study: one between the longitudinal motion of the intima-media complex (input) and the adventitia layer (output) and another between the carotid blood pressure (input) and the longitudinal motion of the intima-media complex (output). In addition to the average transfer functions of the whole population, the same transfer functions were computed separately for the upper and lower quartiles of the data arranged according to the average deviation of the longitudinal motion waveform of the intima-media complex (IO_dev_). Multiple subject specific waveforms have been observed in the longitudinal motion of the common carotid artery (Cinthio et al., 2006; Ahlgren et al., 2012; Yli-Ollila et al., 2013, 2016), thus the impact of the different waveforms on the transfer functions are observed here.

The transfer function analysis was performed separately for every continuous, good quality part of the separate 10-s videos. The length of these segments varied from one successful, heartbeat long motion tracking to 10 s (length of the entire video). First, the trend in the signals was removed using linear detrending and then the signals were windowed using a periodic Hanning window to avoid spectral leakage. Next, the signals, which all had the sampling frequency of 85 Hz, were zero-padded to the same length of 1000 samples to simplify the calculations. The power spectral densities of the input and output signals as well as the cross power spectrum were computed as follows:

(1) $$\begin{array}{l} P_{xx}\left(f\right)=\frac{1}{LF_{s}U}X\left(f\right)X^{*}\left(f\right), \end{array}$$

(2) $$\begin{array}{l} P_{yy}\left(f\right)=\frac{1}{LF_{s}U}Y\left(f\right)Y^{*}\left(f\right), \end{array}$$

(3) $$\begin{array}{l} P_{xy}\left(f\right)=\frac{1}{LF_{s}U}X\left(f\right)Y^{*}\left(f\right), \end{array}$$

where f is frequency, *P*_*xx*_ is the power spectral density of the input, *P*_*yy*_ is the power spectral density of the output, *P*_*xy*_ is the cross power spectrum of the input and output, *X* is the Fourier transform of the windowed input signal, *Y* is the Fourier transform of the windowed output signal, *L* is the length of the corresponding signal, *F*_*s*_ is the sampling frequency, *U* is the energy of the Hanning window ($U=1/L\sum w_{j}^{2}=0.375$, where w_*j*_ is the j:th value of the Hanning window) and * nominates complex conjugate. The time invariant transform function between input and output signals was calculated as follows:

(4) $$\begin{array}{l} TF\left(f\right)=P_{xy}\left(f\right)/P_{xx}\left(f\right), \end{array}$$

which gives transfer function in a complex frequency space. In this study, the widely used Bode plot was used to illustrate the complex results, i.e., transfer functions' amplitude and phase parts are displayed separately in their own graphs and the amplitude part is shown in decibels. Furthermore, since this transfer function computation procedure is made separately for every 1–10 s segments of the longitudinal motion and the blood pressure data, the final presented transfer functions for individual subjects are the averages of the segmental transfer functions.

A magnitude squared coherence function was used to estimate how much of the output signal can be linearly explained by the input signal in different frequencies. The coherence function was computed as:

(5) $$\begin{array}{l} C_{xy}\left(f\right)=\frac{{\left|P_{xy}\left(f\right)\right|}^{2}}{P_{xx}\left(f\right)P_{yy}\left(f\right)}. \end{array}$$

The coherence value over 0.5 was considered to be a significant sign of at least a partly linear relationship between input and output signals.

Amplitude and phase values on the heart rate frequency were measured from each subject's transfer function. This was done by defining a subject specific heartbeat band: the peak of the *P*_*xx*_ of the blood pressure signal of each subject was detected and a 0.5 Hz (± 0.25 Hz) wide band was centered on that peak. The average amplitude and phase values of the transfer function were computed from the heartbeat band, neglecting the values on frequencies where the magnitude squared coherence between input and output was lower than 0.5.

### Stiffness indices

To define the stiffness of the artery, two different stiffness indices were measured: (1) Augmentation index adjusted for the heart rate of 75 beats per minute (Aix@75) was automatically computed from the aortic pressure waveform by the used SphygmoCor applanation tonometry system. (2) Young's elastic modulus (E_*Y*_) was computed from the average diameter curve of the 5-min recording of the carotid artery as follows:

(6) $$\begin{array}{l} E_{Y}=\frac{3\left(1+\frac{A_{avg}}{WCSA}\right)D_{diastole}^{2}\;\times PP_{carotid}}{D_{systole}^{2}-D_{diastole}^{2}}, \end{array}$$

where *A*_*avg*_ is the average lumen area, WCSA is the vessel wall cross-sectional area during diastole, computed from the intima-media thickness by assuming cylindrical geometry. The intima-media thickness measurement was done manually from the first video frame by drawing a line on top of the lumen-intima border and on top of media-adventitia border. *D*_*systole*_ is the systolic diameter, *D*_*diastole*_ is the diastolic diameter and *PP*_*carotid*_ is the pulse pressure in carotid artery.

### Statistical methods

Some of the indices used in this study were not normally distributed and linearity was not expected between every index, thus Spearman's correlation analysis was used to find conformities between the measurements. A *p*-value lower than 0.05 was considered to be statistically significant.

## Results

The average amount of heart cycles per subject that were included from the 5-min recordings into the transfer function analysis was 260, with the range being 132–322. The clinical characteristics and the blood pressures as well as the average heart rates are displayed in Table 1.

**Figure 3:**
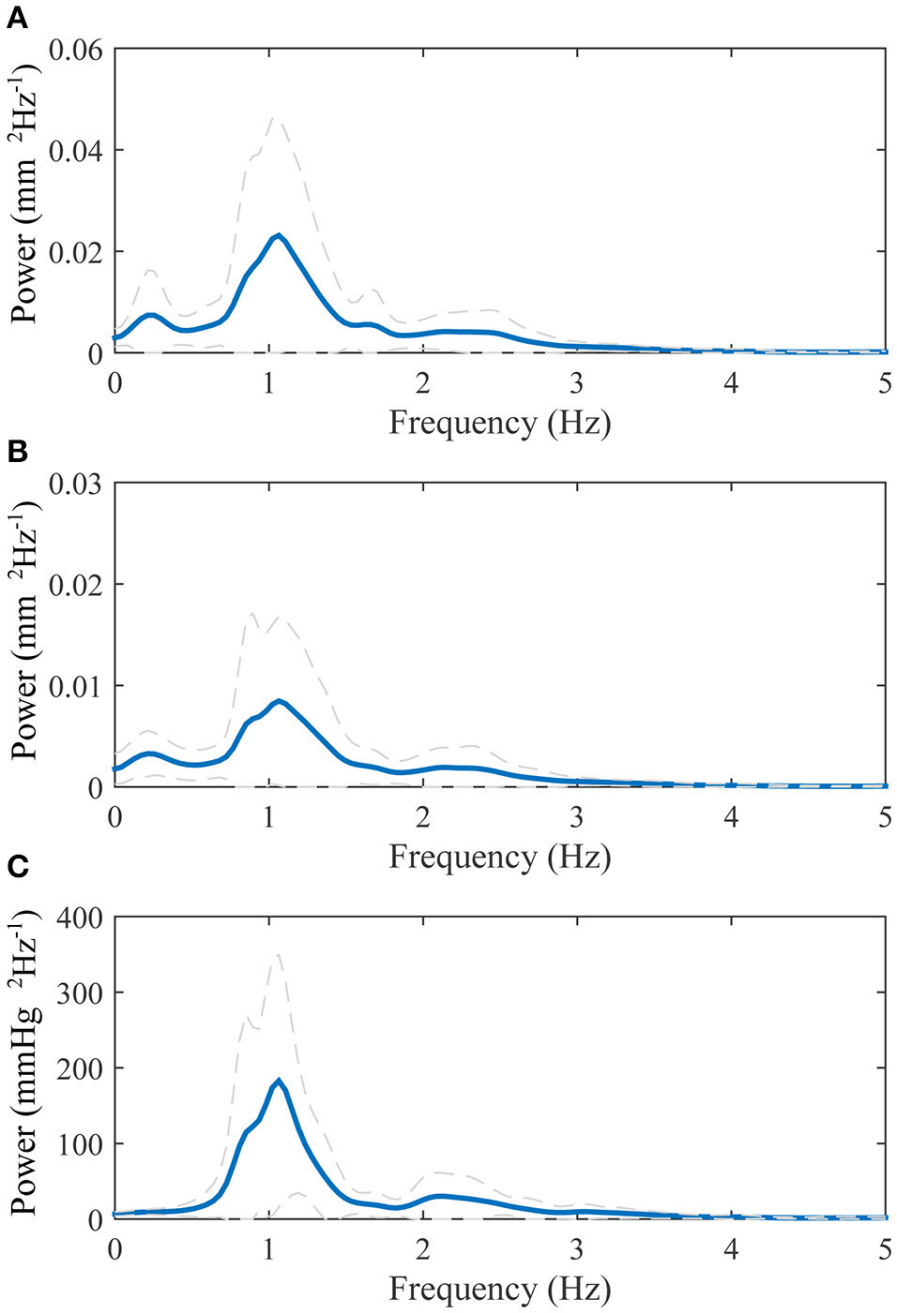
**Group averaged power spectrums: (A)**, longitudinal motion of the intima-media complex; **(B)**, longitudinal motion of the adventitia layer; **(C)**, blood pressure derived from the diameter change curve. The blue thick line displays the mean and the dashed gray lines displays the standard deviation.

The average power spectrums of the longitudinal motion of the intima-media complex and the adventitia layer as well as the blood pressure signal are displayed in Figure 3. The main power in all of the average spectrums is on the band 0–3 Hz, with a large peak on the 1.1 Hz frequency, which is the frequency of the average heart rate within the population. In addition, a peak with low amplitude is visible above 2 Hz frequency in all power spectrums. In the power spectrums of the longitudinal motions, an additional smaller peak is visible on the frequency 0.2 Hz.

**Figure 4:**
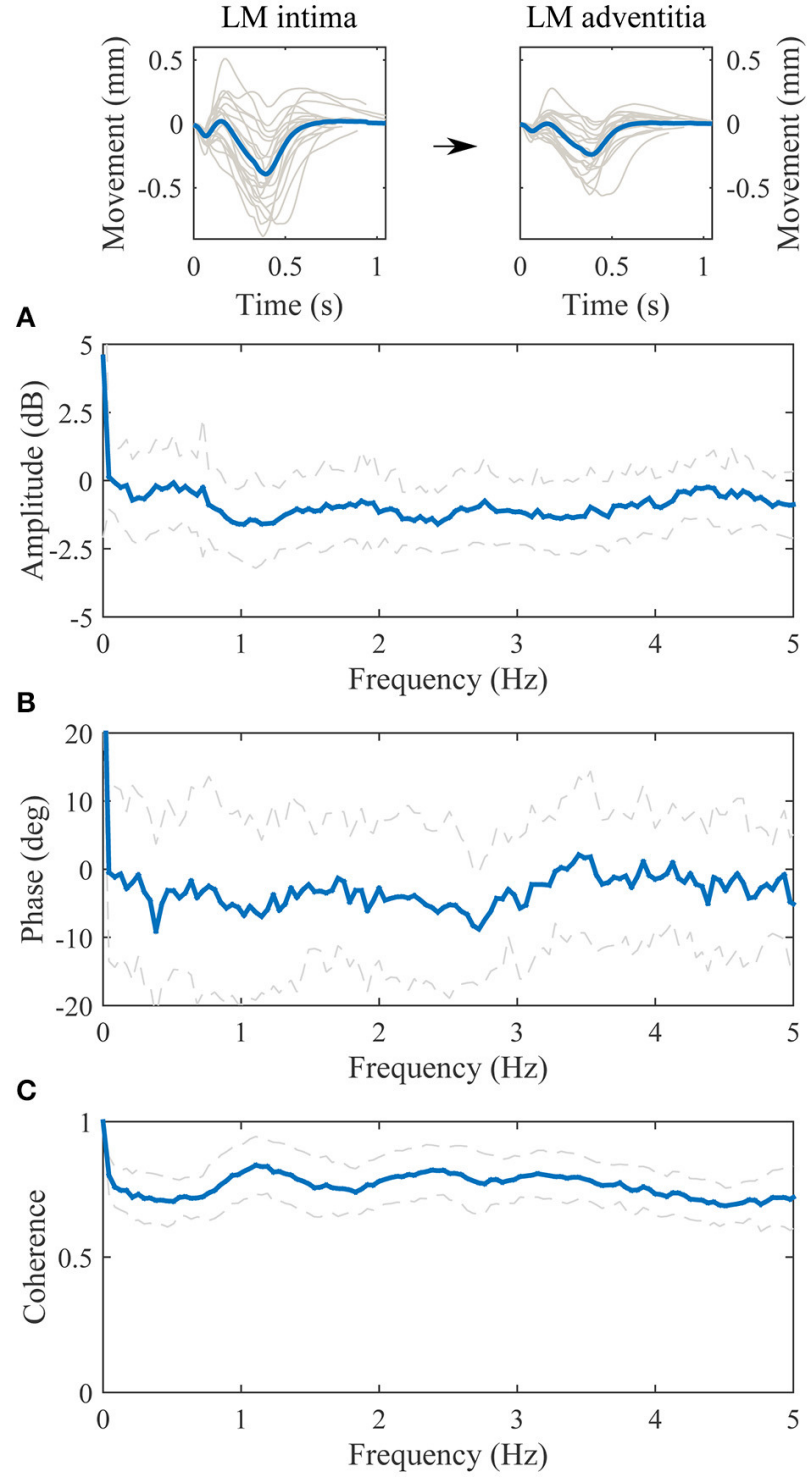
**Group averaged transfer function between the longitudinal motion (LM) of the intima-media complex (input) and the adventitia layer (output). (A)**, The amplitude part of the transfer function; **(B)**, the phase part of the transfer function; **(C)**, the magnitude squared coherence function. On **(A–C)**, the blue line is the average and dashed lines represent the standard deviation. Representative, subject specific average heart-cycle-long input and output signals used in the computation of the transfer function (gray lines) and their averages (blue lines) are presented on the top of the figure.

The transfer function between the longitudinal motion of the intima-media complex and the adventitia layer is displayed in Figure 4. The amplitude part of the transfer function is negative in decibel scale throughout the observed spectrum (0–5 Hz), meaning that the longitudinal motion of the adventitia layer has lower amplitude than the intima-media complex. The maximum attenuation is on the frequency 1.0 Hz, where the kinetic energy of the longitudinal motion of the adventitia layer drops 1.6 dB (31%) compared to the intima-media complex, indicating that 17% of the longitudinal motion amplitude is lost in the transition on the frequency where the most power is in the longitudinal motion signals. The average standard deviation on the 0–5 Hz frequency of the amplitude part of the transfer function is approximately 1.4 dB, indicating a large variation between individuals. The phase part of the transfer function is negative, indicating that the motion of the intima-media complex priors the adventitia layer. On the frequency 1.0 Hz the longitudinal motion of the intima-media complex priors the adventitia layer by 6.8 degrees (18.9 ms). The coherence of the transfer function is well above 0.5 throughout the observed spectrum.

**Figure 5:**
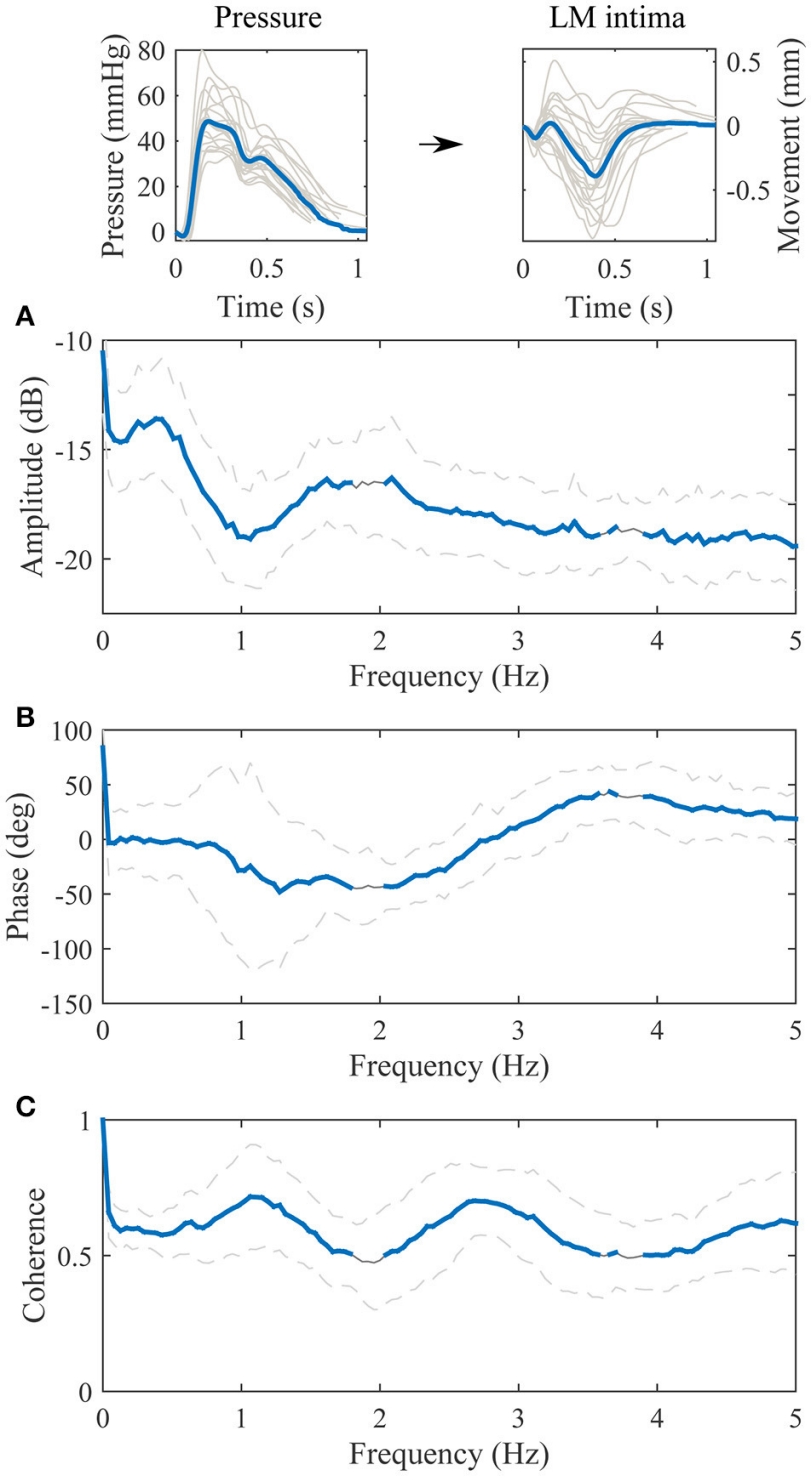
**Group averaged transfer function between the blood pressure [mmHg] (input) and the longitudinal motion (LM) of the intima-media complex [mm] (output). (A)**, The amplitude part of the transfer function; **(B)**, the phase part of the transfer function; **(C)**, the magnitude squared coherence function. On **(A–C)**, the gray line is the average, the gray dashed lines represent the standard deviation and the thick blue line highlights the frequencies of the transfer function where coherence is over 0.5. Representative, subject specific average heart-cycle-long input and output signals used in the computation of the transfer function (gray lines) and their averages (blue lines) are presented on the top of the figure.

The transfer function between the blood pressure and the longitudinal motion of the intima-media complex is displayed in Figure 5. The value of the amplitude part of the transfer function decreases in higher frequencies but there is also an additional, large notch in the amplitude part of the transfer function on the frequency 1.0 Hz. The phase part of the transfer function is negative on the band from 0 to 3 Hz. Although, on the band from 0.5 to 1.5 Hz the variation in the phase spectrum is large; the maximum standard deviation varies between −120 and 70 degrees, which may be due to large variation of the waveform shape of the longitudinal motion curve (antegrade and retrograde oriented movement). The coherence of the transfer function is mainly above 0.5 throughout the spectrum, only merely under 0.5 on the independent frequency bands from 1.8 to 2.0 Hz and from 3.6 to 3.9 Hz.

**Figure 6:**
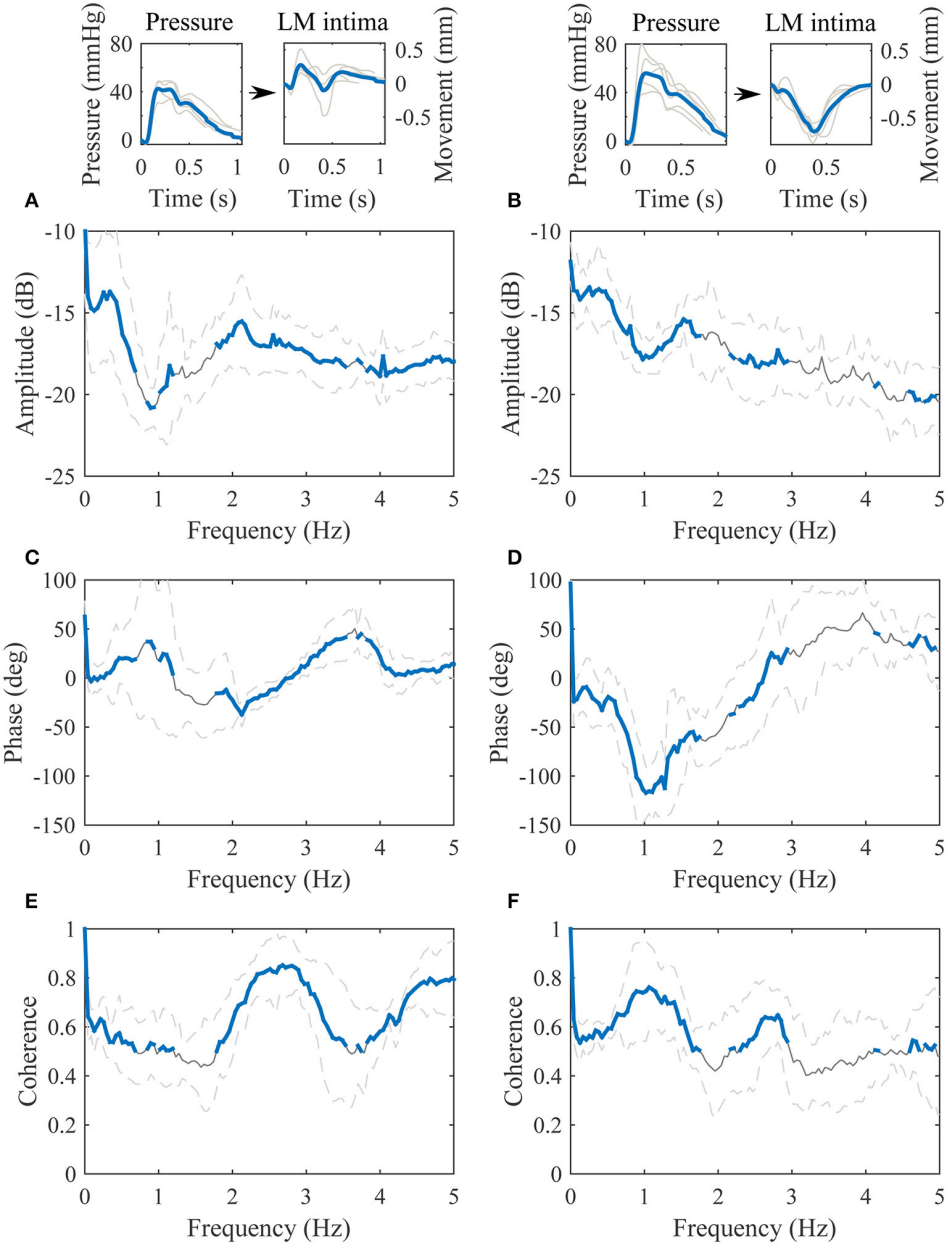
**Transfer functions between the blood pressure [mmHg] (input) and the longitudinal motion (LM) of the intima-media complex [mm] (output)**. The left paragraph represents the upper quartile of the dataset arranged according to the IO_dev_ value and the right paragraph represents the lower quartile. **(A,B)**, the amplitude parts of the transfer functions; **(C,D)**, the phase parts of the transfer functions; **(E,F)**, the magnitude squared coherences. On **(A–F)**, the gray line is the average, the gray dashed lines represent the standard deviation and the thick blue line highlights the frequencies of the transfer functions where coherence is over 0.5. Representative, subject specific average heart-cycle-long input and output signals used in the computation of the transfer functions (gray lines) and their averages (blue lines) are presented on the top of the figure.

The population was divided into quartiles according to the main orientation of the longitudinal waveform (IO_dev_). Thirteen subjects out of 19 had a negative IO_dev_ value i.e., the longitudinal motion was retrograde oriented. The transfer functions between the blood pressure and the longitudinal motion of the intima-media complex, displaying the antegrade (upper quartile) and retrograde (lower quartile) oriented waveforms, respectively, are presented in Figure 6. The amplitude parts of the quartile transfer functions are fairly similar to the corresponding whole population transfer function. The clearest difference between the transfer functions obtained from the antegrade and retrograde oriented waveforms is the phase part around 1 Hz: the antegrade transfer function has clear positive phase (peak at 37 degrees) and the retrograde transfer function has a clear negative phase (peak at −117 degrees). Another observable difference between the quartile transfer functions is the location of the maximum coherence: in the antegrade oriented population the maximum coherence is between 2 and 3 Hz whereas in the retrograde oriented population it is on 1 Hz and there is only a smaller peak on the 2–3 Hz band.

The correlations of the amplitude and the phase values on the individual heartbeat band and the arterial stiffness measures are presented in Table 2. The amplitude and the phase values of the transfer function between the longitudinal motion of the intima-media complex and the adventitia layer did not display any correlation to known arterial stiffness indices. On the other hand, the phase part of the transfer function between the blood pressure and the longitudinal motion of the intima-media complex had a clear, indirect correlation with Aix@75 (*p* < 0.01) and *E*_*Y*_ (*p* < 0.05).

**Table 2:** **Correlations between the transfer function parameters defined from the heartbeat band and arterial stiffness indices as well as longitudinal amplitude parameters**.

	**Aix@75**	***E***_***Y***_	**IO_ampl_**	**IO_ante_**	**IO_retro_**	**IO_dev_**
**TF: INTIMA-MEDIA -**> **ADVENTITIA**						
Amplitude of the heartbeat band	0.245	0.139	−0.411	−0.072	−0.270	0.209
Phase of the heartbeat band	0.048	0.221	0.214	−0.221	0.226	−0.144
**TF: BLOOD PRESSURE -**> **INTIMA-MEDIA**						
Amplitude of the heartbeat band	−0.033	−0.160	0.833^***^	−0.490^*^	0.886^***^	−0.730^***^
Phase of the heartbeat band	−0.658^**^	−0.495^*^	−0.379	0.432	−0.443	0.560^*^

*TF, transfer function; Aix@75, augmentation index adjusted for the heart rate of 75 beats per minute; E_Y_, Young's elastic modulus; IO_ampl_, peak-to-peak amplitude of the longitudinal motion of the intima-media complex; IO_ante_, anterage component of the longitudinal amplitude; IO_retro_, retrograde component of the longitudinal amplitude; IO_dev_, average deviation in longitudinal direction during a heart cycle*.

* *p < 0.051*,

** *p < 0.01*,

*** *p < 0.001*.

There were no significant correlations between the referential arterial stiffness measures (Aix@75 and E_*Y*_) and the longitudinal motion parameters IO_ampl_ (*r* = −0.013, *p* = 0.957; *r* = −0.023, *p* = 0.926), IO_ante_ (*r* = −0.023, *p* = 0.926; *r* = −0.481, *p* = 0.037), IO_retro_ (*r* = 0.026, *p* = 0.915; *r* = 0.088, *p* = 0.721), and IO_dev_ (*r* = −0.065, *p* = 0.792; *r* = −0.319, *p* = 0.183), respectively.

## Discussion

There are three primary findings in the present study. (1) In a healthy population, the longitudinal motion of the carotid artery wall occurs first in the intima-media complex and is then followed by the longitudinal motion of the adventitia layer. The delay between the motions is 6.8 degrees (~18.9 ms) and the attenuation is 1.6 dB in the heartbeat frequency (1.0 Hz). (2) The blood pressure (or the diameter change) in the common carotid artery has a partly linear relationship to the longitudinal motion of the intima-media complex and is thus a potential initiating force for the longitudinal motion. (3) The phase difference between the blood pressure signal and the longitudinal motion of the intima-media complex on the heartbeat band has a potential relationship to arterial stiffness.

The power spectrums of the longitudinal motion and the blood pressure signal both display a peak on the frequency 1.1 Hz, which also is the measured average heart rate frequency within the study population. Since the work of the heart muscle is the origin for the blood pressure and blood flow in arteries, it is logical that most of the power within the longitudinal motion of the common carotid artery wall is on the same frequency band that the heart muscle operates. In addition, a lower peak is visible in the power spectrums above the 2 Hz frequency. This reflects the multiphase nature of the blood pressure and longitudinal motion signals during a cardiac cycle, such as the change of direction of the longitudinal motion during systole. In the power spectrums of the longitudinal motion of the intima-media complex and the adventitia layer, there is also a smaller peak visible on the 0.2 Hz frequency. The effect of breathing to the longitudinal wall motion has been demonstrated previously (Cinthio et al., 2005). The peak in the power spectrums on the frequency 0.2 Hz, presented here, is on the band 0.2–0.33 Hz where the free breathing occurs (Ball et al., 2014) and thus is possibly confirming observation that breathing modifies the longitudinal motion of the common carotid artery wall, although breathing was not directly measured in this study.

Our results show that the longitudinal motion of the common carotid artery wall is first occurring in the intima-media complex and is then followed by the longitudinal motion of the adventitia layer. The average delay between the longitudinal motions is 6.8 degrees on the 1.0 Hz frequency, which translates into 18.9 ms. The media layer is connected to the adventitia by an external elastic lamina which is composed of condensed sheets of elastic fibers (Stevens and Lowe, 1997), thus it is possible that the elastic fibers drag the adventitia layer along, as the intima-media complex moves in the longitudinal direction. According to the amplitude part of the transfer function, 31% (1.6 dB) of the longitudinal kinetic energy of the intima-media complex is lost to drag the adventitia layer along. Since the power (energy) of the longitudinal motion signal is related to the square of the longitudinal amplitude, the 31% lost in power equals to 17% lost in longitudinal motion amplitude. The abatement of the longitudinal motion occurs throughout the observed spectrum (0–5 Hz) and it reaches its maximum on the 1.0 Hz frequency. The standard deviation in the amplitude part of the transfer function between the longitudinal motion of the intima-media complex and the adventitia layer is on average 1.4 dB between frequencies 0 Hz and 5 Hz, meaning that there is quite large variation in the abatement of the longitudinal wall motion between individuals. Since the amplitude of the longitudinal carotid wall motions have been previously connected to arterial stiffness (Zahnd et al., 2011a; Taivainen et al., 2015; Yli-Ollila et al., 2015), this deviation observed in the transfer functions could be related to arterial stiffness but according to our results with 19 healthy individuals, such connection to known arterial stiffness parameters was not found. The possible connection to arterial stiffness should be studied with a larger population, including unhealthy subjects.

The diameter change of the common carotid artery behaves similarly as the blood pressure signal within the artery (Barnett et al., 1961; Patel et al., 1963; Sugawara et al., 2000; Giannattasio et al., 2008) and thus the diameter change was used here as a base to model the blood pressure and to define the transfer function between the blood pressure and the longitudinal motion of the intima-media complex. The coherence between the blood pressure signal and the longitudinal wall motion of the carotid is mainly above 0.5. This is a sign of a partly linear relationship between the signals. According to our results, the amplitude part of the transfer function decreases with higher frequencies and with an additional notch on the 1.0 Hz frequency. This decreasing amplitude within higher frequencies and the notch on the 1 Hz frequency is observed for both antegrade and retrograde oriented wall motions. The phase of the transfer function on the band from 0.5 to 1.5 Hz has large variation within the study population and it displays a clear correlation to previously known arterial stiffness parameters. The correlation between the IO_dev_ and the phase value of the transfer function in the heartbeat band is 0.560. This is due to the large variance in the main direction of the longitudinal motion among the individuals. A positive 1 Hz blood pressure signal and a simultaneous negative (retrograde oriented) 1 Hz longitudinal motion causes approximately 180 degree phase shift into the transfer function and if the longitudinal motion is positive (antegrade oriented) the phase shift is closer to 0 degrees. The phase difference on the frequency band can also be seen in the transfer functions of the lower and upper quartile of the population, arranged according to IO_dev_ value. The antegrade oriented quartile has a positive phase and the retrograde oriented quartile has a negative phase on the band. Nevertheless, the main direction does not explain the phase in the heartbeat band completely but some additional phase difference is also occurring before the longitudinal motion achieves its absolute peak value. This additional phase difference seems to be related to the early arterial stiffening because the phase values in the heartbeat band of the transfer function display a clear correlation to the known stiffness indices but the IO_dev_ does not. This is in line with our previous finding that the shape of the waveform of the longitudinal motion is associated with early stiffening in the common carotid artery (Yli-Ollila et al., 2016).

The higher coherence on the frequencies between 2 and 3 Hz observed in the transfer function of the upper quartile of the data arranged according to the IO_dev_ value is a possible indirect sign of a biphasic longitudinal motion. The coherence on the lower quartile transfer function was significantly lower compared to the upper quartile transfer function on the 2–3 Hz band. The difference can also be seen in Figure 6 where the waveforms with a positive IO_dev_ value oscillate more backwards and forwards than the waveforms with a negative IO_dev_ value. The difference in the coherence is clear but its connection to the biphasic longitudinal motion requires further studies. What is common for all the longitudinal motion waveforms is that they all have a tendency to steer toward the antegrade direction when the blood pressure reaches its maximum, although the amplitude of the antegrade peak in the motion waveform varies a lot. Another similarity with all the waveforms is the steer toward the retrograde direction roughly when the diastolic phase of the heart cycle begins. Similar observations have been made elsewhere (Cinthio et al., 2006; Au et al., 2016).

When interpreting our results one must remember that this is mainly a methodological study presenting a new method to be used for studying longitudinal wall kinetics and cardiovascular diseases, but for clinical use the method needs to be validated with a large and carefully characterized study population including unhealthy subjects. Another notable fact is that the transfer function only describes the linear time-invariant relationship between the measured input and output, i.e., between the longitudinal motion of the intima-media complex and the adventitia layer or between the blood pressure and the longitudinal motion of the intima-media complex. This means that there can possibly be an additional nonlinear relationship between the signals that cannot be observed here. In biological systems, nonlinear input-output characteristics are always present but the coherence functions presented here; serve as evidence that the relationships between the longitudinal motion of the intima-media complex and adventitia layer and between the blood pressure and the longitudinal motion of the intima-media complex are mainly linear. In addition to the nonlinearity, there are other reasons for the lower coherence, such as noise in the measurement signal, especially in higher frequencies. However, in this study the power of the frequencies higher than 5 Hz was negligible. The output can also be due to multiple inputs, which is the most likely reason for the lower coherence in the transfer function between the blood pressure and the longitudinal motion of the intima-media.

## Conclusion

This study describes for the first time how the energy from the blood pressure transfers into the longitudinal motion of the innermost carotid wall layer and from there into the longitudinal motion of the outermost wall layer. Also, the early arterial stiffening seems to modify the phase difference between the carotid blood pressure and the longitudinal motion of the common carotid artery wall. The results give new insights for deriving the driving force of the longitudinal wall motion, for better understanding of the longitudinal wall kinetics and for developing novel methods to detect the early signs of arterial stiffening. Our study population was small and healthy and thus a larger clinical validation is needed to confirm the tentative findings and to study how the transfer functions are modified by different cardiovascular diseases.

## Ethics statement

Fully informed written consent was obtained from each participant and the Ethics Committee of the University of Eastern Finland and Kuopio University Hospital approved the used study protocol.

## Author contributions

HY, MT, TPL, and TML designed the experiment and HY, TPL, and TML acquired the data. HY, MT, TPL, and TML contributed to the analysis and interpretation of data. HY, MT, TPL, and TML participated in the elaboration of the manuscript and gave the final approval for submission and publication, being accountable for all aspects of the present work.

## Funding

We acknowledge the financial support of the Kuopio University Hospital (EVO 5031320, 5031316 and VTR 5031356) and the University of Eastern Finland. In addition, we would like to express our gratitude toward the following foundations for financial support: Science Foundation of Kuopio University Hospital, Aarne and Aili Turunen Foundation, Foundation for the Promotion of Technological Advances, Aleksanteri Mikkonen Foundation, Finnish Foundation for Cardiovascular Research and Antti and Tyyne Soininen Foundation.

### Conflict of interest statement

The authors declare that the research was conducted in the absence of any commercial or financial relationships that could be construed as a potential conflict of interest.
